# Evaluating the Effectiveness of Soft Tissue Therapy in the Treatment of Disorders and Postoperative Conditions of the Knee Joint—A Systematic Review

**DOI:** 10.3390/jcm10245944

**Published:** 2021-12-18

**Authors:** Alicja Jurecka, Maciej Papież, Paulina Skucińska, Artur Gądek

**Affiliations:** 1 Department of Orthopedics and Physiotherapy, Faculty of Health Sciences, Jagiellonian University Medical College, 30-688 Krakow, Poland; artur.gadek@uj.edu.pl; 2 Department of Physiotherapy, Emirates Specialty Hospital, Dubai 505240, United Arab Emirates; maciekpapiez@wp.pl; 3 Students’ Scientific Society, Faculty of Health Sciences, Jagiellonian University Medical College, 31-008 Krakow, Poland; paulina.skucinska@alumni.uj.edu.pl

**Keywords:** fascial manipulation, myofascial release, manual therapy, muscular stretching, musculoskeletal disorders, lower limb, pain

## Abstract

The term “soft tissue therapy” (STT) refers to mechanical methods of treatment involving passive kneading, pressing and stretching of pathologically tense tissues in supporting the process of recovery after surgery or trauma to the musculoskeletal system. The objective of this study was to review current scientific reports evaluating the effectiveness of the use of STT in patients with diseases or after surgical procedures of the knee joint. A systematic search of the popular scientific databases PubMed, Scopus and Embase was performed from inception to 15 October 2021. Eight articles met eligibility criteria and were included in the review. Six papers were related to disorders of the knee joint, while the remaining two studies were related to dysfunctions associated with the conditions after surgical intervention. The findings presented confirmed the effectiveness of STT in orthopaedic patients who showed an increase in lower limb functional parameters. The research has shown that the use of various methods of STT has a significant impact on increasing muscle activity and flexibility as well as increasing the range of motion in the knee joint. The physiotherapeutic methods used had a significant impact on reducing pain and increasing physical function and quality of life. The techniques used reduced the time to descend stairs in patients with knee osteoarthritis. This review summarises the effectiveness of STT as an important form of treatment for orthopaedic patients with various knee joint dysfunctions.

## 1. Introduction

The tissue that constitutes a specific kind of bond, simultaneously co-creating all joint structures, is the connective tissue [1,2]. The active and passive stabilizers of the knee joint are closely interconnected in the fascial system, which is the soft tissue component of the connective tissue system [3]. The joint capsule of the knee joint is directly influenced by the tendons that attach to it, the gastrocnemius, biceps femoris (BF), and semimembranosus muscles [3], as well as more distant structures such as the gluteus maximus muscle, tensor fascia lata, and the iliotibial band [4]. The muscles of the lower limb connect to the deep fascia by means of so-called fibrous expansion or indirect insertions in the muscle fibers (muscular insertion) [5]. The fibrous expansions of the quadriceps running from the medial and lateral gastrocnemius muscle fibers, through the anterior part of the patella, connect to the fascia lata of the thigh and participate in the formation of the patellar retinaculum. The semimembranosus muscle tendon, on the other hand, forms two branches; the first connects to the posterior part of the joint capsule, forming the oblique popliteal ligament, while the second connects to the fascia of the popliteus muscle [5,6]. Changes and damage to the fascial system will therefore play an important role for both the stability of the knee joint as well as the formation of movement restrictions and sensomotor disorders [7,8,9].

Researchers do not agree on one general definition of fascia. However, it is undeniable that it forms a three-dimensional structure with many interdependent layers, located at different depths, from the level of the skin to the periosteum [10]. Fascia is a tissue of our body that is involved in a number of processes, including pathological ones [11]. This is because it is a metabolically active, vascularized and innervated structure, containing both myelinated and non-myelinated nerve fibers and Schwann cells [12]. The deep layers of the muscular fascia and the tendons are richly supplied with small diameter afferent nerve fibers that can conduct pain stimuli [13,14,15]. Autonomic nerve fibers have also been shown to be present in the deep fascia [16,17].

The deep fascia has long been considered as a source of pain, resulting from changes in its structure (densification and/or fibrosis), causing deformation of nerve endings immersed in the fascia [18,19]. The hypothesis that the fascia is directly involved in the pain mechanism is supported by experiments using hypertonic saline. After injecting the deep fascia of the dorsal extensor muscle and the thoracolumbar fascia with hypertonic saline (5.8%), subjects reported the onset of acute pain. The findings indicate that the thoracolumbar fascia is the most sensitive structure to chemical stimulation and as such is likely to be the main cause of the development of non-specific back pain [20,21]; on the other hand, they suggest that sensitization of fascial nociceptors may play a significant role in the pathophysiology of chronic musculoskeletal pain. The authors also demonstrated that sensitized free nerve endings in the muscle fascia continue to be stimulated when the fascia is prestretched by muscle contraction [21].

Disorders of the fascia structure and its properties can therefore affect function and recovery in chronic pain patients [22]. Loss of the physiological property of fascial expansion, impaired gliding of collagen layers in relation to each other and increased friction between fascial layers, often associated with the presence of high levels of abnormal hyaluronic acid (HA) molecules, have been implicated as causes of chronic pain [1,5]. This is particularly important in the knee joint, where smooth tissue movement is particularly important during the concentric and eccentric contraction phases that occur during each step [23]. The result of reduced tissue gliding is increased tissue tension and stiffness, which can lead to overload and repetitive micro-injuries. These, in turn, if left untreated, can impinge on the development of inflammation [24], resulting in pain at rest, adhesions, and in the long term, degenerative changes in tissues and even tissue damage [25]. The described loose connective tissue gliding disorders may also occur secondarily, as a consequence of trauma, surgery, or overload syndrome [1]. It has also been shown that the absolute majority of free nerve endings are very superficially distributed in the fascia and that it contains sympathetic fibers, which may be related to vasoconstriction and ischemic pain [26].

Changes in fascicle innervation occur as a consequence of pathological changes. It has been demonstrated that patients with patellofemoral conflict and anterior compartment knee pain have proliferation of nociceptive fibers (in the pathomechanim of nerve ingrowth) and immunoreaction to substance P within the shortened, compressed lateral knee retinaculum [27]. The referenced findings support clinical observations indicating that retinaculum pathology may play a key role in primary patellofemoral pain as a consequence of increased neural growth factor generation, which is certainly not without impact on knee joint function [1]. Changes in both the histological structure (presence of inflammation and microcalcifications) and the extent of innervation of the tensor fascia lata (nerve fibers atrophy) have been observed in patients with chronic lumbago [28]. This observation may somewhat explain the impaired stability and proprioception at the knee joint [29].

Research in recent years has shown that fascia plays a significant role in muscle force transmission [30,31,32,33], wound healing, skin vascularization, and tropism [34]. Impaired muscle mechanical coordination, proprioception, balance, the occurrence of myofascial pain, and spasms are most commonly associated with dysfunctions of the deep fascia and the epimysium [1]. Changes in the mechanical properties of the fascia may therefore reduce muscle extensibility, generating a disturbance in joint range of motion (ROM) [35]. It is likely that a reduction in the extensibility, or flexibility of the fascial network results in impaired neuromuscular control and fiber recruitment patterns of these muscles [36,37,38]. Alteration of muscle fiber recruitment patterns can lead to changes in the forces transmitted to the connective tissue, which in turn can lead to remodeling of the structure of other connective tissue such as ligaments and the joint capsule [39,40,41]. Thus, over time, changing movement patterns can increase the amount of connective tissue adhesions, leading to reduced mobility, especially in the presence of inflammation and pain [19].

In addition to the functions mentioned above, the fascia is also involved in the process of interoception and proprioception. The process by which the body senses, interprets, integrates and regulates signals coming from within, including from deeper layers of connective tissue, in the context of structural damage to the knee joint, appears to be particularly important [42]. Proprioception is dependent on the functioning of rapidly-adapting specialized mechanoreceptors, which are located in joint capsules and tendons [43]. Analyzing the structure of the fascia, the retinaculum is the most innervated part, rich in free nerve endings, Ruffini’s, Pacini’s, Golgi-Mazzoni’s and less numerous spherical clubs. The retinaculum therefore act as specialized proprioceptive organs [1].

Manual therapy (MT) protects soft tissues against fibrosis induced by overload [44], supports post-trauma (surgery) recovery processes, influences the stimulation of satellite cell proliferation following muscle fiber damage, guarantees the correct course of the inflammatory process, while at the core of its action lies the possibility of modelling the composition of the HA-rich matrix [45,46]. The term “soft tissue therapy” (STT) includes mechanical treatment methods involving passive kneading, pressing and stretching (manual stretching) of pathologically tense tissues with the help of a physiotherapist’s hands (hands-on technique) or an instrument (instrument-assisted soft tissue mobilization, IASTM). Popular methods used in STT include, among others: trigger point therapy, muscle energy technique, skin rolling, and massage therapy [47]. Methods allowing modelling of the fascia structure seem to be particularly important in STT. The myofascial restrictions and perceived lack of glide between the layers of fascia can be reduced through fascial manipulation (FM), myofascial release (MFR), the Graston technique containing IASTM, or deep tissue massage [5,22].

Among the joints of the lower limb, the knee joint is the most exposed to pathological loads generated as a result of joint restrictions or structural disorders of the musculo-fascial bands [48,49,50,51,52]. Research shows that ROM restriction at both the hip and ankle has a significant impact on the risk of injury and degenerative changes in the knee joint [50,51,52,53,54,55]. The balance of tension within the kinematic chain appears to be important in both the prevention of injury, disease, and the treatment of knee joint dysfunctions or postoperative conditions [56,57]. The efficacy of STT has already been proven [44,45,46], and it plays an important role in the physiotherapy of orthopedic patients. Knee osteoarthritis (KO), ligamentous and meniscus damage are among the most common pathologies [58,59] which have a significant impact not only on the biomechanics of the knee joint itself, but of the entire motor system. In the case of structural disorders of the knee joint, a decrease in ROM, muscle strength, and gait speed is observed, as well as an increase in the load transferred through the joint [49]. Common surgical treatments for the knee, such as total knee replacement (TKA) or ligament reconstruction, aim to improve the function of the lower limb. However, there are a number of postoperative conditions, mainly pain, limited ROM, and decreased muscle strength that significantly limit activities and reduce the quality of life of patients [60,61,62,63].

The scientific literature lacks a review paper on the impact of the use of STT as a method potentially leading to the improvement of the functional state of patients with knee joint dysfunctions. The objective of this study was to review the current scientific literature evaluating the effects of STT in patients with medical conditions or after surgical procedures of the knee joint.

## 2. Materials and Methods

This paper is a systematic review of the literature and is structured according to the Preferred Reporting Items for Systematic Reviews and Meta-Analyses (PRISMA) guidelines and statement [64].

### 2.1. Search Strategies and Data Source

A detailed search of popular scientific databases was performed: MEDLINE (PubMed), Scopus and Embase, from inception to 15 October 2021. After identifying and selecting relevant question words based on a preliminary literature review, the authors collaboratively developed a search strategy that was the same for each database. For the PubMed search process, the “Advanced Search” option for “All Fields” was used and screening was manual. Details are shown in Table 1 and Table 2.

### 2.2. Eligibility Criteria

The inclusion criteria were as follows: (1) full-text original research (not study protocols, clinical commentaries or conference proceedings) published in a peer-reviewed scientific journal, (2) article written in English, (3) studies conducted on a human population, (4) history of knee injury or condition in an orthopedic patient, and (5) a soft tissue physiotherapy method used as the sole treatment for pain and soft tissue dysfunction.

Meta-analyses and review articles were rejected in the qualification process. Studies based on the use of complex methods of the patient’s improvement, which did not allow us to unequivocally determine the effectiveness of the soft tissue physiotherapy method, were excluded from the review. Case reports were also not eligible for review.

The criteria for the qualification of articles did not include the date of publication, design and methodological quality of the study.

### 2.3. Data Extraction

At each stage of the search, all studies were checked for eligibility by the lead author (A.J.) and independently verified by the others (A.G., M.P. and P.S.). Data extraction was performed using a standard Microsoft Excel spreadsheet form. The relevant columns of the spreadsheet contained the following information: the names of the authors of the article, the title and type of study, a description of the study group population, the assessment tools used, details of the treatment methods and techniques carried out, and the main findings.

### 2.4. Quality Appraisal

The methodological quality of the studies included in the review, as well as the reliability, relevance and risk of bias of the results obtained were assessed using the Joanna Briggs Institute critical appraisal tools [65]. Author A.J. conducted a critical appraisal of the studies eligible for review, which was then verified by the other authors (A.G., M.P. and P.S.) working independently. Any disagreements between authors were resolved through discussion.

### 2.5. Data Analysis and Synthesis

The authors conducted a detailed analysis and synthesis of the study results using a narrative text approach to summarize the findings on the effectiveness of STT in the treatment of disorders and postoperative conditions of the knee joint. Due to a methodological heterogeneity of studies selected for the review, no statistical analysis between the obtained results have been made.

## 3. Results

### 3.1. Search Strategy Results

**Figure 1:**
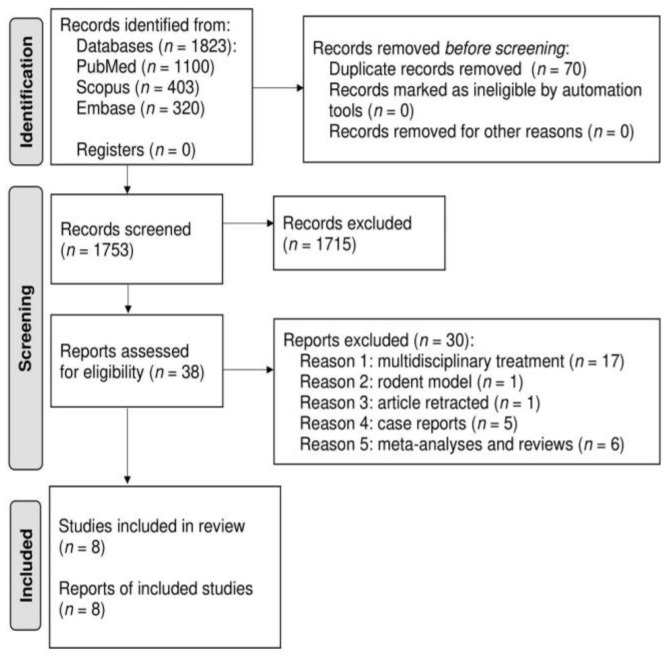
PRISMA flow diagram for search strategy results and eligibility criteria.

The sum of search results in all three electronic journal databases was 1823 articles. After rejecting duplicates, 1753 studies were subjected to the qualification process. Finally, eight papers met the inclusion criteria for the review [66,67,68,69,70,71,72,73]. Details are presented in the PRISMA flow diagram (Figure 1).

The studies included a total of 228 participants. Of the eight studies, two were related to knee dysfunctions associated with status following surgical intervention [66,71], and six were related to knee joint disorders [67,68,69,70,72].

### 3.2. Quality Appraisal Findings

Among four randomized controlled trials included in the review, one article (25%) [69] was of very good quality, and three articles (75%) [66,67,68] were of good quality. Of the three non-randomized experimental studies, one article (33%) [70] was of very good quality, and two articles (67%) [71,72] were of good quality. One case series included in the review was of poor quality [73]. The characteristics and critical appraisal of the studies are presented in Table 3, Table 4, Table 5 and Table 6.

### 3.3. Objective Methods for Evaluating the Effects of the Applied Therapy

#### 3.3.1. Skeletal Muscle Activity

The authors used surface electromyography (sEMG) to measure muscle activity [67,71]. In a study by Cruz-Montecinos et al. [67] it was observed that there was a significant reduction in the activity of the vastus lateralis (VL) following STT. In addition, there was a significant increase in co-contraction for the biceps femoris (BF) and the VL [67]. On the other hand, the results of E Silva et al. [71] testified that after the applied treatment, using the MFR, the activity of the BF and the rectus femoris (RF) significantly increased.

#### 3.3.2. Knee Joint Functional Assessment

Functional assessment of the knee joint was performed using the following measurements and tests: ROM [66,70,71], measurement of flexibility of the hamstring muscles determined by knee extension angle [73], joint position sense (JPS) proprioception test performed with the Orthyo System [68] and stair descent test measured in seconds [71].

Range of motion was the most commonly assessed parameter of knee joint function. The results of the studies [66,70,71] proved that the applied STT techniques significantly increased the ROM of the knee joint. In addition, Winslow et al. [73] have shown that after applying soft tissue mobilization techniques, the flexibility of the hamstring and iliotibial band significantly increased. On the other hand, in a study by Goślińska et al. [68] it was observed that after applying patella mobilization and deep tissue massage, proprioception of the knee joint deteriorated significantly compared to the group of subjects who performed only synergistic and equivalent exercises in a closed kinematic chain. In the aforementioned study by Cruz-Montecinos et al. [67] it was reported that the use of STT had a significant effect on reducing stair descent time in patients with knee osteoarthritis.

### 3.4. Subjective Methods for Assessing Patient Condition

#### 3.4.1. Pain

Pain was the most frequently assessed parameter of the effectiveness of the applied treatment methods. The authors of the studies used the following questionnaires to assess pain: the Numeric Pain Rating Scale [66,67,69,73] and the Visual Analog Scale [68,70,71,72]. The results of the studies [66,67,68,69,70,71,72,73] proved that the applied STT techniques significantly reduced knee joint pain. However, a study by E Silva et al. [71] reported that two patients had no change in pain after MFR and one patient reported worsening pain.

#### 3.4.2. Physical Function and Quality of Life

Physical function and quality of life were measured using the following questionnaires: Western Ontario and McMaster Universities Osteoarthritis Index [66,67,68], Global Rating of Change Scale [73], Lower Extremity Functional Scale [69,73] and Short Form-12 Mental Components [66]. In a study by Winslow et al. [73], improvements in patients’ general condition scores were observed after physiotherapy treatment using a soft tissue mobilization technique. The authors of the studies [66,68] proved that after the application of MT there was a significant reduction in the results of subscales: pain, stiffness, and function [66,68]. The results of a number of studies included in the review [69,73] confirmed that the use of different methods of STT had a significant effect on increasing physical function scores. Additionally, the study by Argut et al. [66] observed that the use of MT had a significant effect on increasing the overall quality of life score.

## 4. Discussion

This review aims to present the effects of STT in the treatment of disease and post-operative injury conditions. The presented research results have confirmed the effectiveness of STT in orthopedic patients in many knee joint dysfunctions related to the conditions following surgical intervention [66,71], as well as knee joint disorders [67,68,69,70,72,73]. Studies have shown that the use of various STT methods has a significant effect on normalizing muscle activity [67,71] and flexibility [73], increasing the ROM in the knee joint [66,68,71]. Reduced pain [66,67,68,69,70,71,72,73] is associated with improved physical function [69,73] and quality of life [66]. The great diversity both in terms of the study population, the STT methods used, and the way the effects of therapy were assessed made it impossible to create direct comparisons of the results obtained by the authors.

### 4.1. Knee Joint Disorders

Among the papers shortlisted for review, the largest group consisted of studies involving people with various knee conditions [67,68,69,70,72,73]. One of these studies described a case series [73], and five presented research papers [67,68,69,70,72], three of which were randomized trials [67,68,69], and two were non-randomized experimental studies [70,72]. The patients included in the studies were diagnosed with the following disease entities: degenerative changes [67,68], arthropathy of the knee joint in the course of hemophilia [70], patellofemoral pain syndrome [69], patellar tendinopathy [72], and lateral compartment syndrome of the knee joint [73]. The predominant STT methods used were: FM including centre of coordination (CC points) [72], muscular stretching of the lower limb [67], myofascial therapy including: superficial sliding anterior and posterior part of the leg [70], deep tissue massage [68], MFR applied to the RF and tensor fascia lata muscle, and iliotibial band [69], STT including the iliotibial band, VL, BF, distal end of the hamstring, and gastrocnemius [73]. In the studies of Cruz-Montecinos et al. [67] and Goślińska et al. [68], the following were further applied: joint mobilizations and/or muscular stretching and/or deep tissue massage, which appear to significantly support the soft tissue healing process. Changes to characteristic areas of the deep fascia (densifications), in addition to joint pain, can cause joint locking. If it is a local change in the structure of the fascia that has arisen in the recent past (fresh densification) then mobilization of the joint may be applicable to soft tissue treatment, because by unblocking the joint the painful afferent is reduced and the pathological tension of the musculoskeletal unit is reduced [74].

A reduction in perceived pain [67,68,69,70,72,73], as well as an improvement in physical function [69,73] was observed in all examined patients with knee joint disorders. Donoso-Ubeda et al. [70] further showed an improvement in joint ROM, while Winslow et al. [73] showed an improvement in the flexibility of the musculo-fascial bands. Cruz-Montecinos et al. [67], on the other hand, observed an improvement in the co-contraction of the thigh muscles: the VL/BF, which seems to be dependent on the level of pain. After therapy, the authors [67] also observed a decrease in the activity of the VL muscle. The decrease in VL activity (with a concomitant increase in lateral contraction) may be related to an improvement in the alignment (decrease in inclination) of the patella, which would be closely linked to the tension of the lateral patellar retinaculum and the iliotibial tract [75,76]. The increase in co-contraction of VL and BF muscles generates the lower knee adduction moment during the support phase, thus allowing a better distribution of loads acting on the knee joint [77]. The use of STT in KO patients has therefore been shown to improve the biomechanics of the knee joint [67]. In a randomized study [68] conducted in patients with knee osteoarthritis, in addition to a number of beneficial changes, a deterioration in proprioception assessed by the JPS test was also demonstrated as a result of STT (MT including: patella mobilization and deep tissue massage). However, it is worth noting the short period of therapy (10 days), after which the measurements were immediately repeated, not allowing the tissues to remodel and adapt receptor excitability to the new, changed loading conditions.

### 4.2. Postoperative Conditions of the Knee Joint

Papers treating the use of STT methods in patients after surgery were also eligible for this review. Two papers dealt with subjects after TKA [66,71], of which only one presented a randomized trial [66]. Numerous STT modalities have been used to treat patients after surgery, such as soft tissue mobilizations, friction massages [66], and MFR [71]. Also, joint mobilization was additionally used in one study [66]. In a randomized trial in patients after TKA, strengthening and stretching of the lower limb muscles and functional exercises were used together with STT [66]. However, it was possible to determine the effect of the MT programme due to the use of a control group with the same exercises as the study group but without the MT programme. In the aforementioned study local cryotherapy was also used to reduce swelling and pain in acute patients. This treatment preceded the application of STT and had no direct effect on the course of treatment [66].

In all papers where the study group were patients after TKA, significant improvement in ROM was noted [66,71]. In two studies that examined pain intensity before and after therapy, most patients reported a significant reduction in pain after treatment [66,71]. One study reported improvements in knee joint function and quality of life [66]. Additionally, only one paper used sEMG as a research tool [71], in which the results showed an increase in muscle activity: the BF and RF muscles after treatment with MFR as monotherapy.

### 4.3. Strengths and Limitations of the Review

The strength of this article is that it is a review of current scientific reports consisting of publications from the last 12 years. This paper consists of studies conducted on a total of 228 orthopedic patients. Many authors used objective assessment methods [66,67,70,71,73]. All studies have confirmed the effectiveness of various techniques being a part of STT in the treatment process of patients with knee joint disorders, as well as after injuries and surgical procedures.

Potential limitations of this review are the small number of randomized controlled trials and the methodological heterogeneity of the studies. Additionally, only papers published in a peer-reviewed journal were included in the review and grey literature was discarded, which may have had the effect of increasing the risk of study bias. Another limitation could be that the authors of all papers [66,67,68,69,70,71,72,73] used subjective assessment methods, which could also have potentially increased the risk of response bias and systematic bias. Due to the methodological heterogeneity of the studies, the review authors did not attempt a meta-analysis.

## 5. Conclusions

This review summarizes the effectiveness of STT as an important form of treatment for orthopedic patients in various knee joint dysfunctions. A particularly significant effect of STT has been demonstrated in reducing pain, as well as normalizing muscle activity and flexibility and increasing mobility of the knee joint. Improving functional parameters of the lower limbs seems to be particularly important to increase physical function and quality of life in patients with medical conditions after trauma or surgery.

## 6. Practical Application

According to the results of the presented studies, it seems appropriate to use STT as a form of treatment for orthopedic patients with diseases and after surgical procedures of the knee joint. The physiotherapy treatment protocol should include STT both as the sole form of treatment and in combination with other methods of treatment, including physical therapy and exercise.

In the future, more studies should be performed, mainly randomized controlled trials, involving a significant number of patients with musculoskeletal dysfunctions, with particular emphasis on a control group. This will provide sufficiently reliable scientific evidence on the effectiveness of STT. Studies should be based on standardized and objective assessment methods in order to exclude the potential risk of measurement error.

## Figures and Tables

**Table 1:** Search criteria based on the PICO model.

PICO	Description
Population	Orthopaedic patients (with dysfunctions of knee joint due to injury, disease or after surgery), children and adults of both sexes
Intervention	Treatment process involved in using a soft tissue physiotherapy method
Comparison	Period before and after applied treatment process
Outcomes	Effects of applied physiotherapeutic methods include, but are not limited to, improvement in knee joint function (including an increase in active and/or passive range of motion), an increase in muscle activity and strength, a reduction in excessive muscle tone and pain

**Table 2:** Summary of search strategies in PubMed, Scopus and Embase databases.

Search—Database	Query Combination
#1—PubMed #2—Scopus #3—Embase	“fascial manipulation” OR “fascial mobilization” OR “fascial therapy” OR “soft tissue manipulation” OR “soft tissue mobilization” OR “soft tissue therapy” AND “pain”
#4—Summary of search results	#1 AND #2 AND #3

**Table 3:** Study characteristics.

Author, Year	Participant Characteristics				Interventions/Number of Treatment Sessions	Outcome Measures	Main Findings (Pre vs. after the Treatment)
	*n*	*n* F:M	Age (Years)	Diagnosis/Patient’s Condition			
Argut, S.K. 2021 [66]	IG = 21; CG = 21	IG = 17:4; CG = 20:1	IG = 69.3 ± 7.4; CG = 67.5 ± 5.01	Total knee arthroplasty	IG: 1. Strengthening and stretching lower limb muscles and functional exercises (transfers, stair climbing); 2. Manual therapy including: patellofemoral and tibiofemoral joint glides, soft tissue mobilizations applied to the medial, lateral and posterior surfaces of the knee, and friction massages; CG: the same exercises as the IG (without manual therapy program); Number of treatment sessions: N/S	NPRS	After the treatment: significant ↓ in pain in IG compared to CG (*p* = 0.001; d = 6.5); pre vs. after two weeks vs. after two months of the treatment: 8.5 ± 0.4 vs. 1.7 ± 0.2 vs. 0.1 ± 0.2 points;
						ROM	Knee flexion after the treatment: significant ↑ in ROM in IG compared to CG (*p* = 0.001; d = 1.4); pre vs. after two weeks vs. after two months of the treatment: 105.2 ± 15.0 vs. 110.2 ± 7.8 vs. 118.5 ± 7.8 degrees;
						WOMAC	After the treatment: significant ↓ in average WOMAC total score (pain, stiffness, function) in IG compared to CG (*p* = 0.006; d = 0.9); pre vs. after two weeks vs. after two months of the treatment: 76.6 ± 11.8 vs. 35.7 ± 12.9 vs. 13.9 ± 5.7 points;
						SF-12 MCS	After the treatment: significant ↑ in the mean value of the total quality of life score in the IG compared to CG (*p* = 0.01; d = 1.1); pre vs. after two weeks vs. after two months of the treatment: 31.2 ± 8.8 vs. 34.7 ± 8.9 vs. 38.8 ± 7.8 points
Cruz-Montecinos, C. 2016 [67]	IG = 8; CG = 8	IG = 8:0; CG = 8:0	IG = 64.37 ± 2.9; CG = 61 ± 1.9	Knee osteoarthritis	IG: soft tissue therapy including: muscular stretching of the psoas iliacus, hamstring, quadriceps, adductors, gastrocnemius and tensor fascia lata, joint mobilizations, and periarticular band tensing; CG: supine position, hands placed around the patella, without exerting pressure or moving the tissue; Single treatment session	NPRS	After the treatment: significant ↓ in pain in IG compared to (*p* = 0.018); pre vs. after the treatment: 3.5 ± 0.7 vs. 0 ± 0.2 points;
						EMG (sEMG)	After the treatment: significant ↓ in activity of the vastus lateralis muscle in IG compared to CG (*p* = 0.034); pre vs. after the treatment: 2195.14 ± 543.31 vs. 2041.49 ± 568.08; Co-contraction after the treatment: significant ↑ in co-contraction for the biceps femoris and the vastus lateralis muscle in the IG compared to CG (*p* = 0.014); pre vs. after the treatment: 21.38 ± 10.78 vs. 23.76 ± 11.54 V;
						WOMAC	After the treatment: a significant correlation between pain and change in co-contraction for the vastus lateralis muscle was noted in the IG (r = 0.804; *p* = 0.008);
						Stair descent time cycle [s]	After the treatment: significant ↓ in stair descend time in IG compared to CG (*p* = 0.019); pre vs. after the treatment: 3.43 ± 0.72 vs. 3.04 ± 0.07 s
Goślińska, J. 2020 [68]	EG = 27; MG = 27; CG = 27	N/S	EG = 65.0 ± 7.4; MG = 66.1 ± 4.7; CG = 63.0 ± 6.6	Knee osteoarthritis	EG: synergy and balance exercises in a closed kinematic chain; MG: manual therapy including: patella mobilization and deep tissue massage; CG: no intervention (treatment); 10 treatment sessions per 10 days	Joint position sense (Orthyo System)	After the treatment: significant ↑ in values regarding the end angle in the left knee flexion position in MG compared to the other study groups (*p* = 0.004); pre vs. after 10 sessions of the treatment: 67.0 ± 9.1 vs. 72.7 ± 9.5 degrees;
						WOMAC	After the treatment: significant ↓ in average WOMAC total score (pain, stiffness, function) in EG and MG compared to CG (*p* < 0.05); pre vs. after 10 sessions of the treatment: 45.9 ± 13.7 vs. 39.7 ± 12.8 points for EG and 46.3 ± 19.0 vs. 40.1 ± 21.7 points for MG; there was no significant difference between EG and MG;
						VAS	After the treatment: significant ↓ in pain in both lower limbs in EG and MG (*p* < 0.01); there was no significant difference in results between EG and MG
Telles, G. 2016 [69]	MG = 9; EG = 9	N/S	MG = 63.3 ± 12.1 EG = 61.8 ± 17.3;	Patellofemoral pain syndrome	MG: 1. Exercises to strengthen hip muscles, home exercises; 2. Myofascial release applied to the rectus femoris and tensor fascia lata muscle, and iliotibial band; EG: the same exercises as the MG (without myofascial release); 10 treatment sessions per five weeks	NPRS	After the treatment: significant ↓ in pain in MG compared to EG (*p* = 0.01; d = 0.35); pre vs. after the treatment: 6.5 ± 2.6 vs. 3.4 ± 2.8 points;
						LEFS	After the treatment: significant ↑ in physical function score in MG compared to EG (*p* = 0.008; d = 0.30); pre vs. after the treatment: 45.3 ± 15.4 vs. 56.2 ± 14.3 points
Donoso-Ubeda, E. 2018 [70]	IG = 8;CG = 8	N/S	IG = 39 ± 13.02; CG = 42.38 ± 14.15	Hemophilic arthropathy of the knee	IG: myofascial therapy including: superficial sliding anterior and posterior part of the leg combined with active flexion and extension movements of the foot, popliteal fascia, hands crossed technique applied to the anterior compartment of the knee, and degravitation and slight traction of the lower limb; CG: no intervention (treatment); Three treatment sessions per three weeks	ROM	Knee flexion after the treatment: significant ↑ in ROM of both lower limbs in IG compared to CG (*p* < 0.05); pre vs. after the treatment: 107.00 ± 32.44 vs. 110.75 ± 33.83 degrees for the right knee, and 111.63 ± 29.17 vs. 117.25 ± 30.55 degrees for the left knee; Knee extension after the treatment: ignificant ↑ in ROM of the right lower limb in IG compared to CG (*p* = 0.041); pre vs. after the treatment: 5.0 ± 6.50 vs. 3.25 ± 6.04 degrees;
						VAS	After the treatment: significant ↓ in pain of the right lower limb in IG compared to CG (*p* < 0.050); pre vs. after the treatment: 1.25 ± 1.28 vs. 0.75 ± 1.36 points
E Silva, D.C.C.M. 2018 [71]	33	22:11	68.2 ± 7.85	Total knee arthroplasty	Myofascial release including: gluteal fascia, posterior fascia lata, posterior crural fascia, and plantar fascia; Single treatment session	ROM	Knee flexion after the treatment: significant ↑ in ROM (*p* = 0.01); pre vs. after the treatment: 54.7 vs. 60.4 degrees;
						EMG (sEMG)	After the treatment: significant ↑ in activity of the biceps femoris (*p* = 0.037) and the rectus femoris muscle (*p* = 0.167); pre vs. after the treatment: 0.088 ± 0.066 vs. 0.101 ± 0.085 V for the biceps femoris, and 0.077 ± 0.052 vs. 0.083 ± 0.055 V for the rectus femoris muscle;
						VAS	22 study participants reported no pain before treatment; After the treatment: eight patients reported a 56.9% reduction in pain; two patients reported no change in pain; one patient reported an increase in pain
Padrelli, A. 2009 [72]	18	5:13	29.2	Patellar tendinopathy	Fascial manipulation including CC points: AN-GE, ER-GE, IR-GE, LA-GE, ME-GE, and RE-GE; Single treatment session	VAS	After the treatment: significant ↓ in pain (*p* < 0.001); pre vs. after the treatment: 67.8/100 vs. 25.6/100 points
Winslow, J. 2014 [73]	4	4:0	27–43	Lateral knee pain syndrome	Soft tissue mobilization technique including: anterior and posterior border of the iliotibial band, vastus lateralis, biceps femoris, distal end of the hamstring, and gastrocnemius; Three treatment sessions per week for three weeks	KEA	After the treatment: ↑ in flexibility of the hamstring and iliotibial band;
						LEFS	After the treatment: ↑ in physical function score; three athletes who were able to run without pain improved their overall performance by 9–19 points;
						GRCS	After the treatment: ↑ in overall athlete score by three to five points (improved);
						NPRS	After the treatment: ↓ in pain; two athletes reported no pain at all, one athlete rated his pain as 1/10 points; one subject experienced lateral knee pain after running 0.3 miles, which he rated as 9/10 points

*n*—number of participants, f—females, m—males, EG—Exercise Group, IG—Intervention Group, MG—Manual Group, N/S—Not Stated, CC—Centre of Coordination, AN-GE—ante-genu, ER-GE—extra-genu, IR-GE—intra-genu, LA-GE—latero-genu, ME-GE—medio-genu, RE-GE—retro-genu, EMG—Electromyography, sEMG—surface Electromyography, GRCS—Global Rating of Change Scale, KEA—Knee Extension Angle, LEFS—Lower Extremity Functional Scale, NPRS—Numeric Pain Rating Scale, ROM—Range of Motion, SF-12 MCS—Short Form-12 Mental Components, VAS—Visual Analog Scale, WOMAC—Western Ontario and McMaster Universities Osteoarthritis Index, d—effect size, *p*—value, r—correlation coefficient, ↓—decrease, ↑—increase.

**Table 4:** Critical appraisal of randomized controlled trials.

Author, Year	Was True Randomization Used for Assignment of Participants to Treatment Groups?	Was Allocation to Treatment Groups Concealed?	Were Treatment Groups Similar at the Baseline?	Were Participants Blind to Treatment Assignment?	Were Those Delivering Treatment Blind to Treatment Assignment?	Were Outcomes Assessors Blind to Treatment Assignment?	Were Treatment Groups Treated Identically Other Than the Intervention of Interest?	Was Follow up Complete and if not, Were Differences between Groups in Terms of Their Follow up Adequately Described and Analyzed?	Were Participants Analyzed in the Groups to Which They Were Randomized?	Were Outcomes Measured in the Same Way for Treatment Groups?	Were Outcomes Measured in a Reliable Way?	Was Appropriate Statistical Analysis Used?	Was the Trial Design Appropriate, and Any Deviations from the Standard RCT Design (Individual Randomization, Parallel Groups) Accounted for in the Conduct and Analysis of the Trial?	Johanna Briggs Institute Score
Argut, S.K. 2021 [66]	✓	✓	✓	✓	✓	X	✓	✓	✓	✓	✓	✓	✓	12
Cruz-Montecinos, C. 2016 [67]	✓	✓	X	✓	✓	✓	✓	✓	✓	✓	✓	✓	✓	12
Goślińska, J. 2020 [68]	✓	✓	X	✓	✓	✓	✓	✓	✓	✓	✓	✓	✓	12
Telles, G. 2016 [69]	✓	✓	✓	✓	✓	✓	✓	✓	✓	✓	✓	✓	✓	13

✓—Yes, X—No.

**Table 5:** Critical appraisal of non-randomized experimental studies.

Author, Year	Is It Clear in the Study What Is the “Cause” and What Is the “Effect” (i.e., There Is No Confusion about Which Variable Comes First)?	Were the Participants Included in Any Comparisons Similar?	Were the Participants Included in Any Comparisons Receiving Similar Treatment/Care, Other than the Exposure or Intervention of Interest?	Was There a Control Group?	Were There Multiple Measurements of the Outcome Both Pre and Post the Intervention/Exposure?	Was Follow up Complete and If Not, Were Differences between Groups in Terms of Their Follow up Adequately Described and Analyzed?	Were the Outcomes of Participants Included in Any Comparisons Measured in the Same Way?	Were Outcomes Measured in a Reliable Way?	Was Appropriate Statistical Analysis Used?	Johanna Briggs Institute Score
Donoso-Ubeda, E. 2018 [70]	✓	✓	✓	✓	✓	✓	✓	✓	✓	9
E Silva, D.C.C.M. 2018 [71]	✓	✓	✓	X	✓	✓	✓	✓	✓	8
Padrelli, A. 2009 [72]	✓	✓	✓	X	✓	✓	✓	✓	✓	8

✓—Yes, X—No.

**Table 6:** Critical appraisal of case series.

Author, Year	Were There Clear Criteria for Inclusion in the Case Series?	Was the Condition Measured in a Standard, Reliable Way for All Participants Included in the Case Series?	Were Valid Methods Used for Identification of the Condition for All Participants Included in the Case Series?	Did the Case Series Have Consecutive Inclusion of Participants?	Did the Case Series Have Complete Inclusion of Participants?	Was There Clear Reporting of the Demographics of the Participants in the Study?	Was There Clear Reporting of Clinical Information of the Participants?	Were the Outcomes or Follow up Results of Cases Clearly Reported?	Was There Clear Reporting of the Presenting Site(s)/Clinic(s) Demographic Information?	Was Statistical Analysis Appropriate?	Johanna Briggs Institute Score
Winslow, J. 2014 [73]	X	✓	✓	X	X	X	X	✓	X	X	3

✓—Yes, X—No.

## Data Availability

The data are available from the online database or on request from the corresponding author.
